# Supramolecular assemblies of carbon nanocoils and tetraphenylporphyrin derivatives for sensing of catechol and hydroquinone in aqueous solution

**DOI:** 10.1038/s41598-021-84294-7

**Published:** 2021-03-03

**Authors:** Syeda Aqsa Batool Bukhari, Habib Nasir, Lujun Pan, Mehroz Tasawar, Manzar Sohail, Muhammad Shahbaz, Fareha Gul, Effat Sitara

**Affiliations:** 1 Department of Chemistry, School of Natural Sciences (SNS), National University of Sciences and Technology (NUST), Islamabad, H-12 Pakistan; 2 School of Physics, Dalian University of Technology, Dalian, China

**Keywords:** Chemistry, Materials science, Nanoscience and technology

## Abstract

Non-enzymatic electrochemical detection of catechol (CC) and hydroquinone (HQ), the xenobiotic pollutants, was carried out at the surface of novel carbon nanocoils/zinc-tetraphenylporphyrin (CNCs/Zn-TPP) nanocomposite supported on glassy carbon electrode. The synergistic effect of chemoresponsive activity of Zn-TPP and a large surface area and electron transfer ability of CNCs lead to efficient detection of CC and HQ. The nanocomposite was characterized by using FT-IR, UV/vis. spectrophotometer, SEM and energy dispersive X-ray spectroscopy (EDS). Cyclic voltammetry, differential pulse voltammetry (DPV) and electrochemical impedance spectroscopy were used for the electrochemical studies. CNCs/Zn-TPP/GCE nanosensor displayed a limit of detection (LOD), limit of quantification (LOQ) and sensitivity for catechol as 0.9 µM, 3.1 µM and 0.48 µA µM^−1^ cm^−2^, respectively in a concentration range of 25–1500 µM. Similarly, a linear trend in the concentration of hydroquinone detection was observed between 25 and 1500 µM with an LOD, LOQ and sensitivity of 1.5 µM, 5.1 µM and 0.35 µA µM^−1^ cm^−2^, respectively. DPV of binary mixture pictured well resolved peaks with anodic peak potential difference, ∆E_pa(CC-HQ),_ of 110 mV showing efficient sensing of CC and HQ. The developed nanosensor exhibits stability for up to 30 days, better selectivity and good repeatability for eight measurements (4.5% for CC and 5.4% for HQ).

## Introduction

The population explosion and a brisk increase of industrialization over the globe have ensued in intensified release of various detrimental compounds into the ecological environment. These harmful chemical pollutants are destroying the stability of the Earth by adding to the increased toxicology of the environment. Aforementioned emerging pollutants may include unregulated chemical substances such as noxious gases (NOx, SOx), natural toxins, various volatile substances, byproducts produced as a result of disinfecting drinking water, heavy metals, cosmetics, endocrine disruptors, cancer promotors, chemical pigments and dyes, and medicinal products^1–3^. Catechol and hydroquinone, the dihydroxybenzene positional isomers, are being used in different commercialized products that include cosmetics, pesticides, different pharmaceuticals, dyes, leather industry, photography etc. and considered as the xenobiotic pollutants^4–9^. The leaching of catechol and hydroquinone into air and water contributes to environmental pollution. Due to its reduced degradability and toxic profile catechol has been enlisted as pollutant by US Environmental Protection Agency (EPA) and European Union (EU)^10^. Catechol has also been categorized as a Group 2B carcinogen by the International Agency for Research on Cancer (IARC) and its high exposure further leads to severe hypertension and breakdown of the central nervous system^11^. Similarly due to the high toxicity of hydroquinone, its use has been banned in the European cosmetics industries by European Union (EU) legislation and in China the concentration for the emission of hydroquinone is strictly limited to 4.45 × 10^–3^ M^12^. Due to their accumulation in the living organisms, there is a need to detect, remove and monitor such toxic compounds through some convenient method. Generally, catechol and its positional isomer (HQ) co-exist in the ecological environment and their redox peaks overlap leading to difficulty in detection of each isomer^13,14^.

Hence, different electrochemical techniques that are less tedious, easily transportable and simple are extensively employed^15^. In contrast the conventional method of high performance liquid chromatography, phosphorescence^16^, chemiluminescence^6,17^, fluorescence^18^ pH-based flow injection analysis^19^, capillary electrophoretic method^20^ and various solvent extraction techniques^21^ were used previously but their handling requires much sophisticated laboratory setups, highly trained professionals, expensive instruments, tedious analysis procedures which lead to difficulties in real-time analysis.

Porphyrins are the macrocyclic compounds with extensive conjugated structure. Owing to their electron rich system, synthetic versatility and inherent stability, tetraphenylporphyrins and metallated tetraphenylporphyrins are the potential candidates for chemical sensors, solar cells^22^, catalysis, optical sensors^23^, immunosensors^24^, photosensitizers for the cancer detection, and other photoelectrochemical applications. These tetrapyrrolic, ubiquitous macrocycles and their metal derivatives have been exploited to covalently and non-covalently functionalize carbon nanostructures viz graphene sheets and carbon nanotubes etc. to be used in the sensing of different analytes. Similarly, zinc-tetraphenylporphyrin have been used in water splitting application and its composite with reduced graphene oxide has been used for the detection of dopamine due to its high chemical activity and stable co-ordinate bonds of the complex^25,26^.

Glassy carbon electrode (GCE) modified by ferrocene-Nafion, CNTs^27,28^, mesoporous carbon ^29–31^, diamond doped by boron^31,32^, nanodiamond^33^, graphene, graphene aerogel^34^, carbon black^35^ and carbon dots^36^ have been previously employed as modifiers for different analytes e.g. phenolic compounds including catechol and hydroquinone detection etc. and these different kinds of nanomaterials have emerged as potential candidates for electrochemical sensors^37^, adsorbents and further have been employed to resolve environmental pollutants^38^.

Realizing the exceptional conformation and the combination of significant properties i.e. good electrical conductivity and mechanical properties of carbon nanocoils has anticipated the researchers to practice these fascinating coiled polycrystalline-amorphous morphological structures for construction of different functional materials such as nanosensors and other nanoelectronic devices. Depending on the nature of functionalization of carbon nanocoils these candidates have found their potential applications in the fabrication of hydrogen storage materials^39^, electrodes in supercapacitors, electrocatalysts in fuel cells^40^, electrodes in micro/nanosensors or wearable sensors like flexible multifunctional strain sensors to monitor the pulse rate, breathing and the vibrations with efficient sensitivity encompassing the fields of artificial intelligence, electronic tongues, electronic noses and other devices^41–43^, real-time sensors to recognize humidity changes in various physical conditions of the human body and the environment thermal sensors^44^ and also some other commercialization purposes of CNCs based materials^45^ such as electromagnetic transformers and switches^46^ etc. Similarly, in another report carboxylic acid group functionalized CNCs were incorporated into the Quartz Crystal Microbalance (QCM) platform for the sensing of ammonia gas at lower temperature with a detection limit of 50 ppm^47^. Silver nanosheets decorated carbon nanocoils supported on nickel foam free-standing 3D electrode has been fabricated for the non-enzymatic sensing of glucose with higher sensitivity and lower detection limits^48^. The scope of CNCs in the field of chemical sensors is yet to be explored and appeared as an emerging field in electrochemistry.

**Figure 1:**
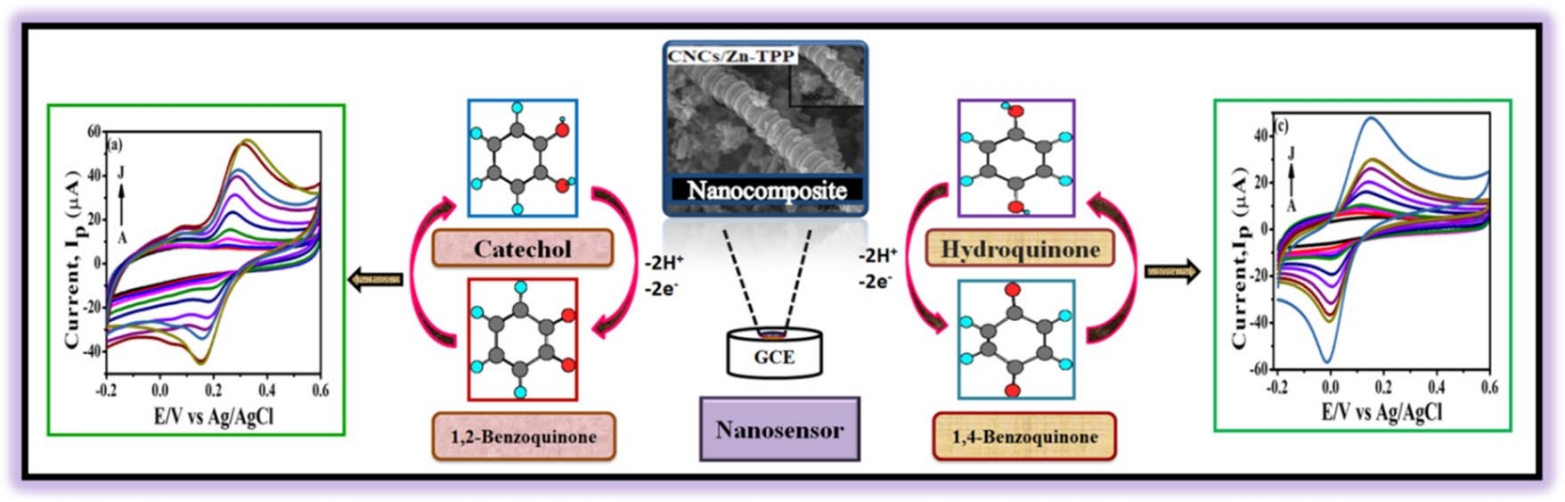
Graphical abstract representing fabrication of sensor and the detection of catechol and hydroquinone.

Herein, the present study is aimed at employing chemoresponsive behavior of Zn-TPP towards development of catechol and hydroquinone sensor. For this application of electrochemical sensing carbon nanocoils (CNCs) were non-covalently functionalized by zinc tetraphenylporphyrin (Zn-TPP) to prepare the CNCs/Zn-TPP nanocomposite for the electrochemical detection of catechol and hydroquinone. To the best of our knowledge CNCs/Zn-TPP nanocomposites have not been used before for the electrochemical detection of catechol and hydroquinone. The graphical representation of the present research work is shown in Fig. 1.

## Results and discussion

### Characterization

**Figure 2:**
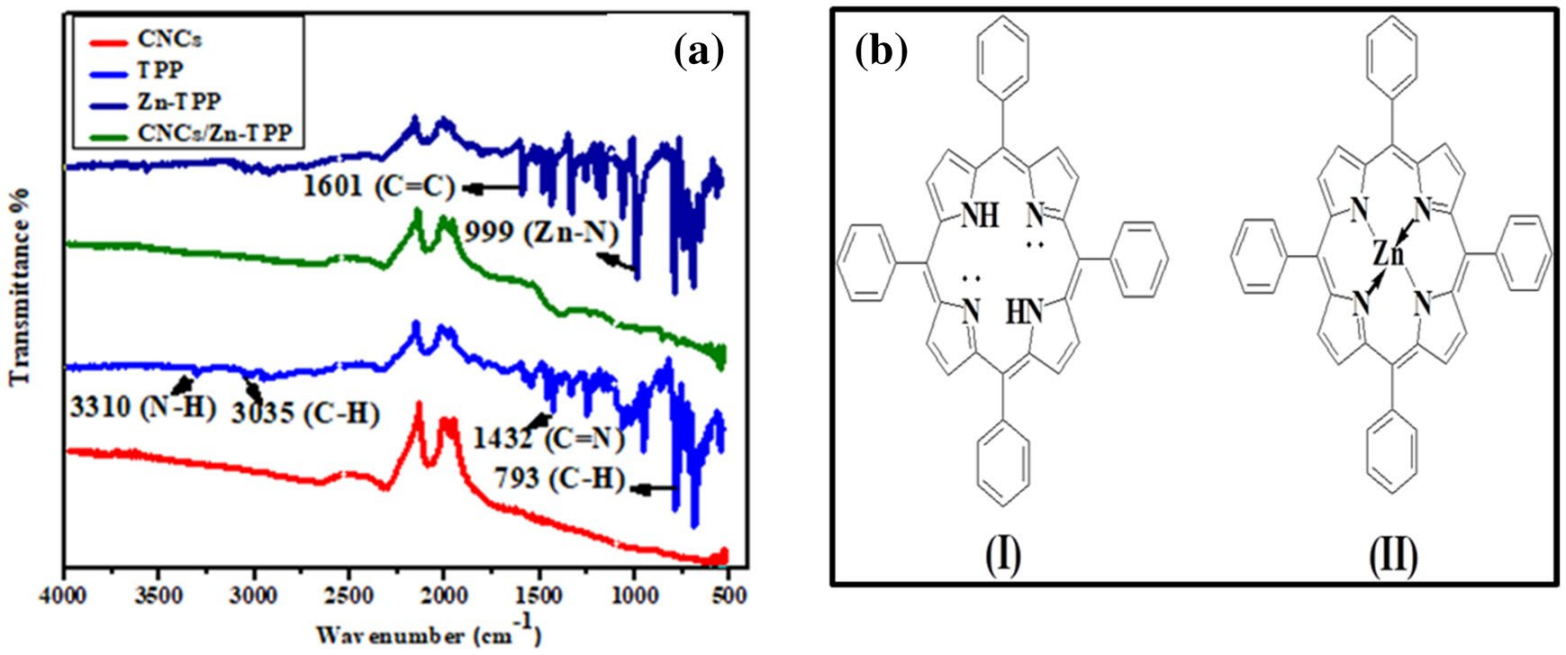
(**a**) FT-IR spectra of CNCs, TPP, Zn-TPP and CNCs/Zn-TPP nanocomposite, (**b**) structure of TPP (I) and Zn-TPP (II).

Figure 2 shows FT-IR spectra of CNCs, TPP, Zn-TPP and CNCs/Zn-TPP. The presence of bands at 3310 cm^−1^ (N–H_stretch_), 3035 cm^−1^ (C–H_stretch_), 1601 cm^−1^ (C=C), 1432 cm^−1^ (C=N) and 739 cm^−1^ (C–H bend) provide the evidence for the formation of TPP^49^. When zinc is incorporated into TPP, the band 3310 cm^−1^ (N–H_stretch_) vanishes whereas a slight shift in all the other bands along with the turn up of a band at 999 cm^−1^ (Zn-N) demonstrates synthesis of Zn-TPP^50^. The molecular structure of TPP and Zn-TPP is shown in Fig. 2 (b). The formation of CNCs/Zn-TPP is suggested by comparing Zn-TPP, CNCs and CNCs/Zn-TPP as all bands in the region from 500 to 2000 cm^−1^ for Zn-TPP are present in the nanocomposite as compared to bare CNCs, but with lower intensity that depicts the formation CNCs/Zn-TPP nanocomposite.

**Figure 3:**
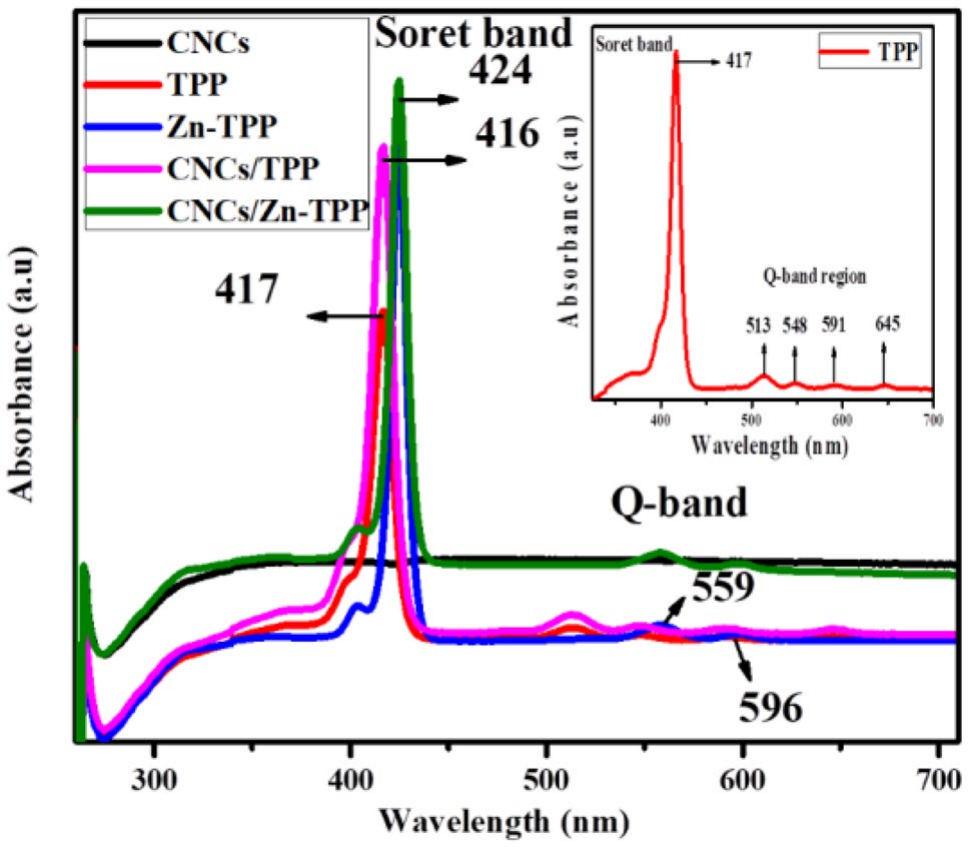
UV/visible spectra of CNCs, TPP, CNCs/TPP, Zn-TPP and CNCs/Zn-TPP in DMF.

Figure 3 illustrates the UV/visible spectra of CNCs, TPP, Zn-TPP, CNCs/TPP and CNCs/Zn-TPP. TPP and Zn-TPP are characterized by presence of Soret bands (400–450 nm) and Q-bands (500–700 nm)^51^. The inset in Fig. 3 displays TPP spectrum with an intense Soret peak at 417 nm with four supplementary Q-bands at 513 nm, 548 nm, 591 nm and 645 nm^26,49,52^. The Soret band (423 nm) of Zn-TPP exhibits bathochromic shift with magnified intensity. The decrement in the number of Q-bands in Zn-TPP is attributed to the reorganization of the structural geometry. TPP possesses planar structure and lies in D_2h_ point group. On introduction of Zn^2+^ into the TPP the configuration of macrocycle transforms to square planar (D_4h_ point group) as the metal co-ordinates with pyrrolic nitrogen atoms decrease of Q-bands occur^52^. A broad band at 316 nm with very low absorption intensity for CNCs appeared due to aromatic system^53^. In CNCs/Zn-TPP absorption intensity increases as compared to pure Zn-TPP due to the non-covalent pi–pi interaction between aromatic system of CNCs and Zn-TPP and the same happens in CNCs/TPP as shown in Fig. 3^54^.

UV/vis. spectroscopic analysis suggests the formation of CNCs/Zn-TPP and CNCs/TPP nanocomposites. A bathochromic shift in the Soret band and an increase in its intensity signifies that the CNCs/Zn-TPP nanocomposite is formed. Similarly, we can also observe a decrease in the number of Q-bands of nanocomposites as compared to the pristine materials. Soret band shifts from 423 nm (Zn-TPP) to 424 nm (CNCs/Zn-TPP) and a hypsochromic shift from 417 nm (TPP) to 416 nm observed in CNCs/TPP but more intensified Soret bands were there in both nanocomposites. CNCs/Zn-TPP showed less intense Q bands with a red shift as compared to pristine Zn-TPP (Fig. 3).

Scanning electron microscope (SEM) was used to evaluate the surface morphology of pure CNCs and CNCs/Zn-TPP nanocomposite. Presence of elements i.e. C and Zn in the energy dispersive spectra (EDS) suggests formation of nanocomposite (Fig. S2, Supplementary Information). On the other hand, the EDS analysis of pristine CNCs displays only the major portion of carbon content (Fig. S1, Supplementary Information).

**Figure 4:**
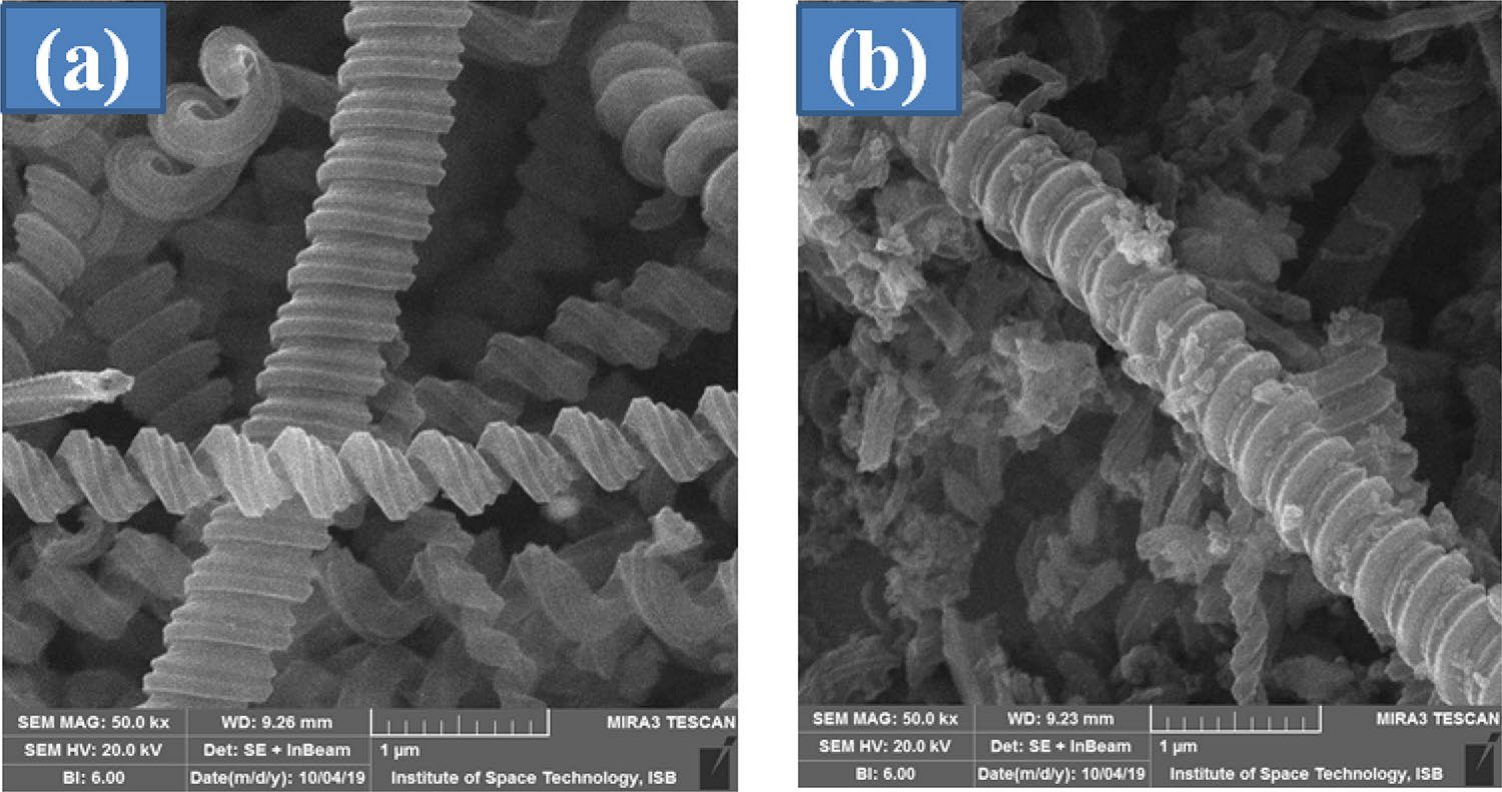
Scanning electron microscopic images of (**a**) pure CNCs and (**b**) CNCs/Zn-TPP.

Figure 4a shows the coiled structure of pure CNCs and Fig. 4b depicts that small Zn-TPP particles are dispersed over the coiled surface. The electrostatic interaction between aromatic pi electrons of TPP and carbon nanocoils keeps the two components of the nanocomposite connected to each other.

### Electrochemical impedance spectroscopy (EIS) of the modified electrodes

**Figure 5:**
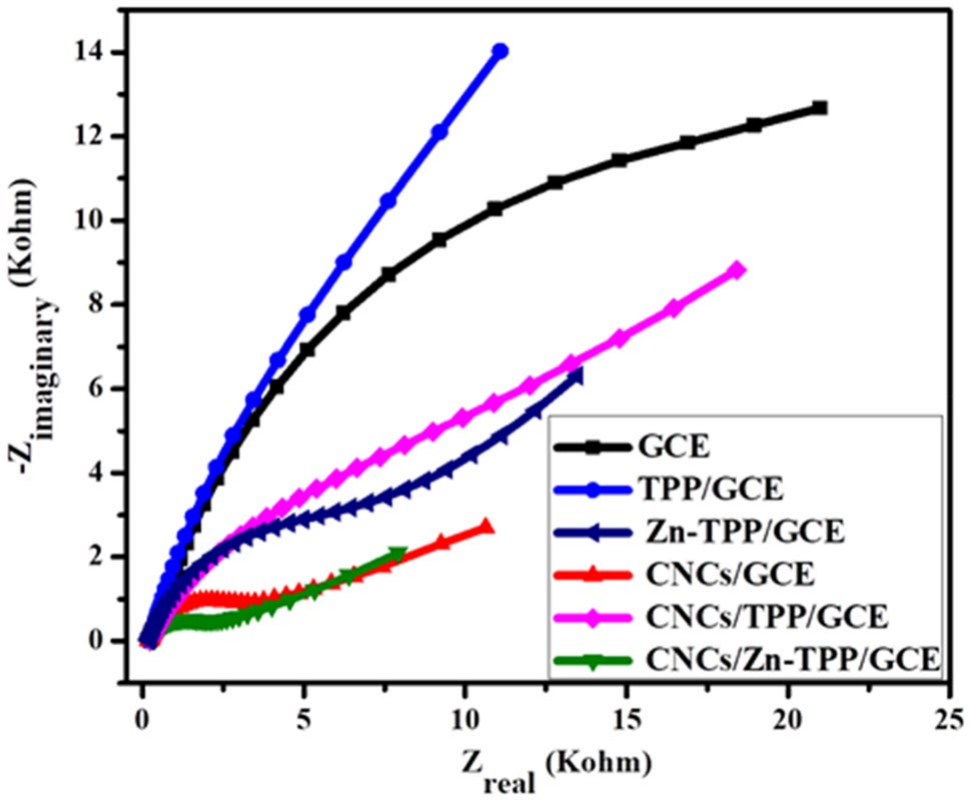
EIS of GCE, TPP/GCE, Zn-TPP/GCE, CNCs/GCE, CNCs/TPP/GCE and CNCs/Zn-TPP/GCE in 0.5 mM K_3_[Fe (CN)_6_] and 0.1 M KCl.

Electrochemical impedance spectroscopy was performed to characterize the interfacial electron transfer properties of modified electrodes. The typical Nyquist plot of EIS represents two regions i.e. semicircle region corresponding to charge transfer resistance (R_ct_) at high frequency range and a linear region that corresponds to diffusion-controlled process at low frequency. Figure 5 illustrates the Nyquist plot for the electrical conductivities of nanosensors and the Randles model was applied for the fitting of data to determine Rct values (Fig. S3, Supplementary Information). The relative conductivities of modified electrodes are as: CNCs/Zn-TPP/GCE (1.7 kΩ) > CNCs/GCE (2.9 kΩ) > CNCs/TPP/GCE (7.0 kΩ) > Zn-TPP/GCE (6.3 kΩ) > TPP/GCE (15.8 kΩ) > GCE (17.9 kΩ). Herein, the R_ct_ values of CNCs/Zn-TPP/GCE, CNCs/GCE and CNCs/TPP/GCE are less than Zn-TPP/GCE, TPP/GCE and bare GCE. This reveals that the incorporation of CNCs enhances the electrical conductivity as it provides the large surface area and electron transfer ability due to conjugated pi electron system. Hence, CNCs/Zn-TPP/GCE exhibits lowest R_ct_ due to the lower interfacial resistance and high conductivity of the nanosensor.

### Electrochemical behavior of modified electrodes towards catechol and hydroquinone

**Figure 6:**
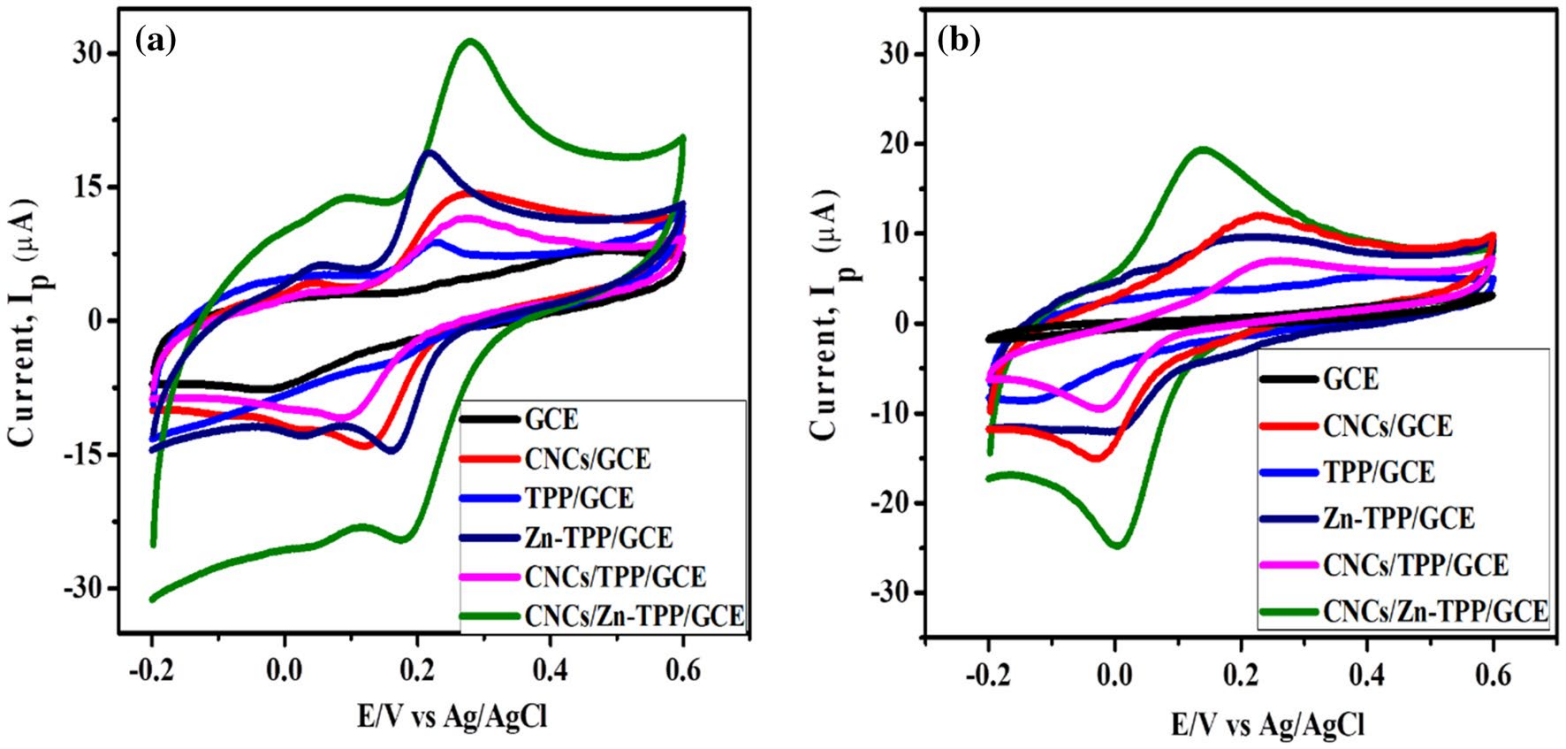
CV of GCE, CNCs/GCE, TPP/GCE, Zn-TPP/GCE, CNCs/TPP/GCE and CNCs/Zn-TPP/GCE in 0.1 M PBS (pH 7) containing (**a**) 500 µM catechol and (**b**) 500 µM hydroquinone.

Cyclic voltammetry was carried out to determine the electrocatalytic behavior of different modified electrodes i.e. GCE, TPP/GCE, Zn-TPP/GCE, CNCs/GCE, CNCs/ TPP/GCE, CNCs/Zn-TPP/GCE towards 500 µM catechol (CC) and 500 µM hydroquinone (HQ) in 0.1 M PBS at sweep rate of 0.05 Vs^−1^. Figure 6a demonstrates the comparison of the peak current and the electrocatalytic behavior of all the electrodes i.e., CNCs/TPP/GCE, Zn-TPP/GCE, CNCS/GCE and TPP/GCE and CNCs/Zn-TPP/GCE in 500 µM CC. CNCs/Zn-TPP/GCE exhibits the highest oxidation peak current (Ipa = 31.25 µA at a peak potential E_pa_ of 0.27 V) as compared to other electrodes. Very weak redox peaks were observed using bare GCE. The elevated charge transfer kinetics of CNCs/Zn-TPP/GCE is due to the synergistic effect of chemoresponsive activity of Zn-TPP, large surface area and electron transfer ability of CNCs.

Similarly, the electrocatalytic response of all the modified electrodes (GCE, TPP/GCE, Zn-TPP/GCE, CNCs/GCE, CNCs/TPP/GCE, CNCs/Zn-TPP/GCE) towards 500 µM hydroquinone is shown in Fig. 6b and here again CNCs/Zn-TPP/GCE exhibited excellent activity to detect HQ. CNCs/Zn-TPP/GCE exhibits an oxidation peak current of 20.3 µA at E_pa_ of 0.13 V for 500 µM HQ and it was the highest current observed as compared to other electrodes.

The electrochemical surface area of the modified electrode CNCs/Zn-TPP/GCE was determined by performing cyclic voltammetry in 0.1 M KCl solution 0.5 mM K_3_[Fe(CN)_6_] and 0.1 M potassium phosphate buffer. Randles–Sevcik equation given below was used to calculate the effective surface area of the electrodes (Table S1, Supplementary Information):

$${I}_{p}=2.69{\times 10}^{5 \, }\times A \times {D}^{1/2} \times {\upsilon }^{1/2} \times {n}^{3/2} \times {C}_{0}$$

where I_p_ designates the peak current in amperes, A = effective surface area (ESA) to be measured, D is the distribution co-efficient of redox probe [Fe(CN)_6_]^3−/4−^ i.e. 7.6 × 10^−6^ cm^2^ s^−1^^55^, ʋ is the scan rate in Vs^−1^, n is the number of electrons (here n equals to 1), C_0_ is the concentration (mol L^−1^) of [Fe(CN)_6_]^3−/4−^.

The ESA of CNCs/Zn-TPP/GCE, CNCs, GCE and Zn-TPP was found to be 0.23, 0.26, 0.19 and 0.07 cm^2^, respectively which indicates that incorporation of CNCs enhances the electrocatalytic surface area of Zn-TPP.

### Effect of pH of the solution

**Figure 7:**
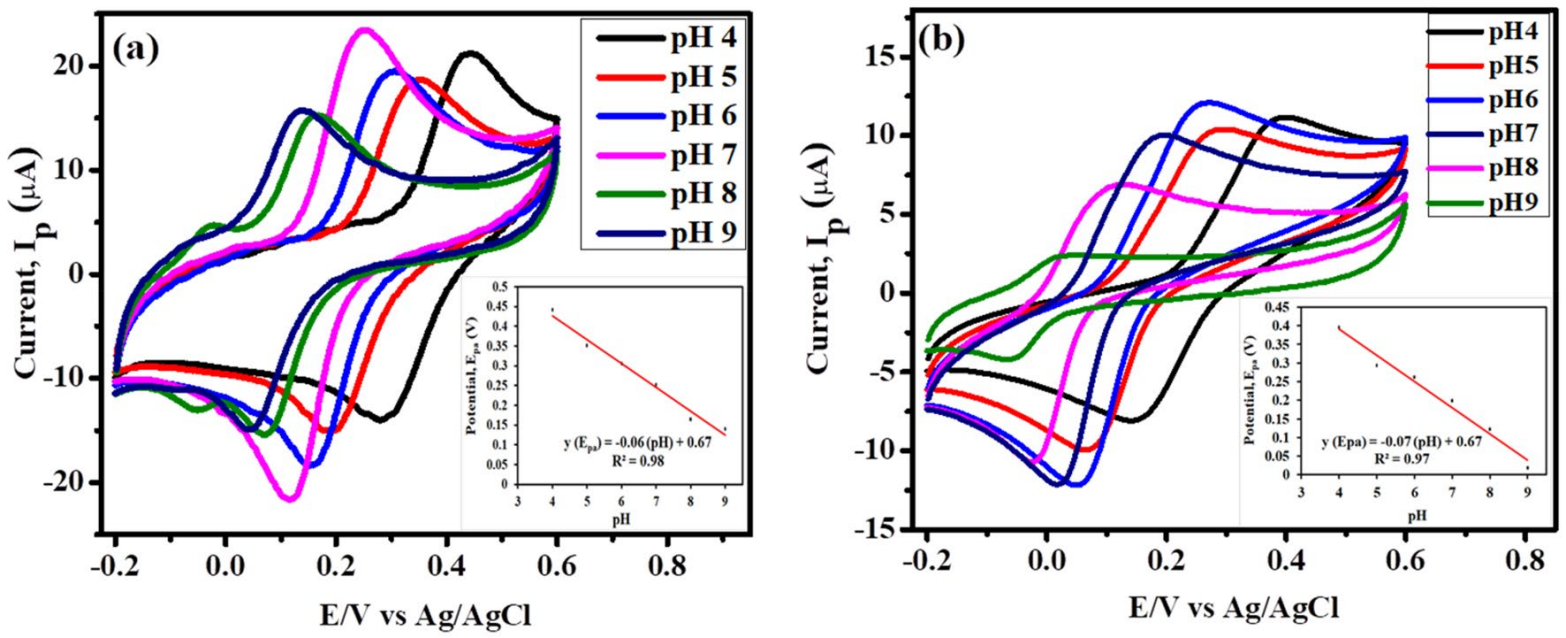
Effect of variable pH (4.0–9.0) solutions of (**a**) 500 µM catechol (**b**) 500 µM hydroquinone (0.1 M PBS) at CNCs/Zn-TPP/GCE electrode. Insets: Calibration plot of E_pa_ vs. pH containing 500 µM CC and HQ.

The pH of the supporting electrolyte is an important factor that influences the behavior of the electrode material during electrochemical studies. In our study we used 0.1 M potassium phosphate buffer to carry out the voltammetric analyses of catechol. The pH was optimized by evaluating the electrocatalytic behavior of the analyte at CNCs/Zn-TPP/GCE electrode with different pH solutions (pH 4–pH 9) of 0.1 M potassium phosphate buffer containing 500 µM catechol. Figure 7a depict that the oxidation peak current of 500 µM CC depends upon the pH of the solution and on increasing the pH the oxidation peak current increases till 7 and decreases from 7 to 9 due to the meager availability of protons in alkaline solution. Also, the redox peaks become prominent on increasing the pH of the solution. At low pH hydroxyl groups of catechol are protonated which decreases its adsorption at the electrode surface due to which decrease in the current is observed^56^.

Further electrochemical studies were done at pH 7 and taken as the optimum. We selected pH 7 as optimal also for further electrocatalytic studies of HQ in contrast to CC due to the detection at lower potential. On increasing the pH of the catechol solution, the shift in the oxidation and reduction peak potentials towards negative and more positive values respectively was observed. It illustrates that the participation of protons takes place in electrode reaction. The linear regression equation for CC obtained from the calibration curve between anodic peak potential and pH is E_pa_ (CC) = -0.06 pH + 0.67 (R^2^ = 0.98).

Figure 7b depicts that the oxidation peak current of 500 µM HQ also depends upon the pH of the solution and the anodic peak current increases till pH 6 to pH 7 and then decreases onwards to pH 9. From the inset in Fig. 7b we can say that as the pH increases the anodic potential shifts towards negative potential showing the participation of protons in electrochemical redox mechanism of hydroquinone. The linear regression equations for HQ obtained from the calibration curve between anodic peak potential and pH is E_pa_ (HQ) = − 0.07 pH + 0.67 (R^2^ = 0.97), as shown in Fig. 7b.

Herein, the values of slopes in Fig. 7a,b are closed to 0.059 V pH^−1^ (theoretical value for Nernst equation and the shift in the oxidation peak potentials with respect to Nernst equation explains the electro-oxidation of CC and HQ is a two protons and two electrons system)^57,58^. It is concluded that the equal number of electrons and protons are playing part in electrochemical redox process of catechol and hydroquinone at the CNCs/Zn-TPP/GCE. The probable mechanism for the electrochemical detection of CC and HQ is given in Fig. S4, which shows that the electrochemical detection of CC and HQ is a coupled two-electron reversible process.

### Effect of variable scan rate (ʋ)

**Figure 8:**
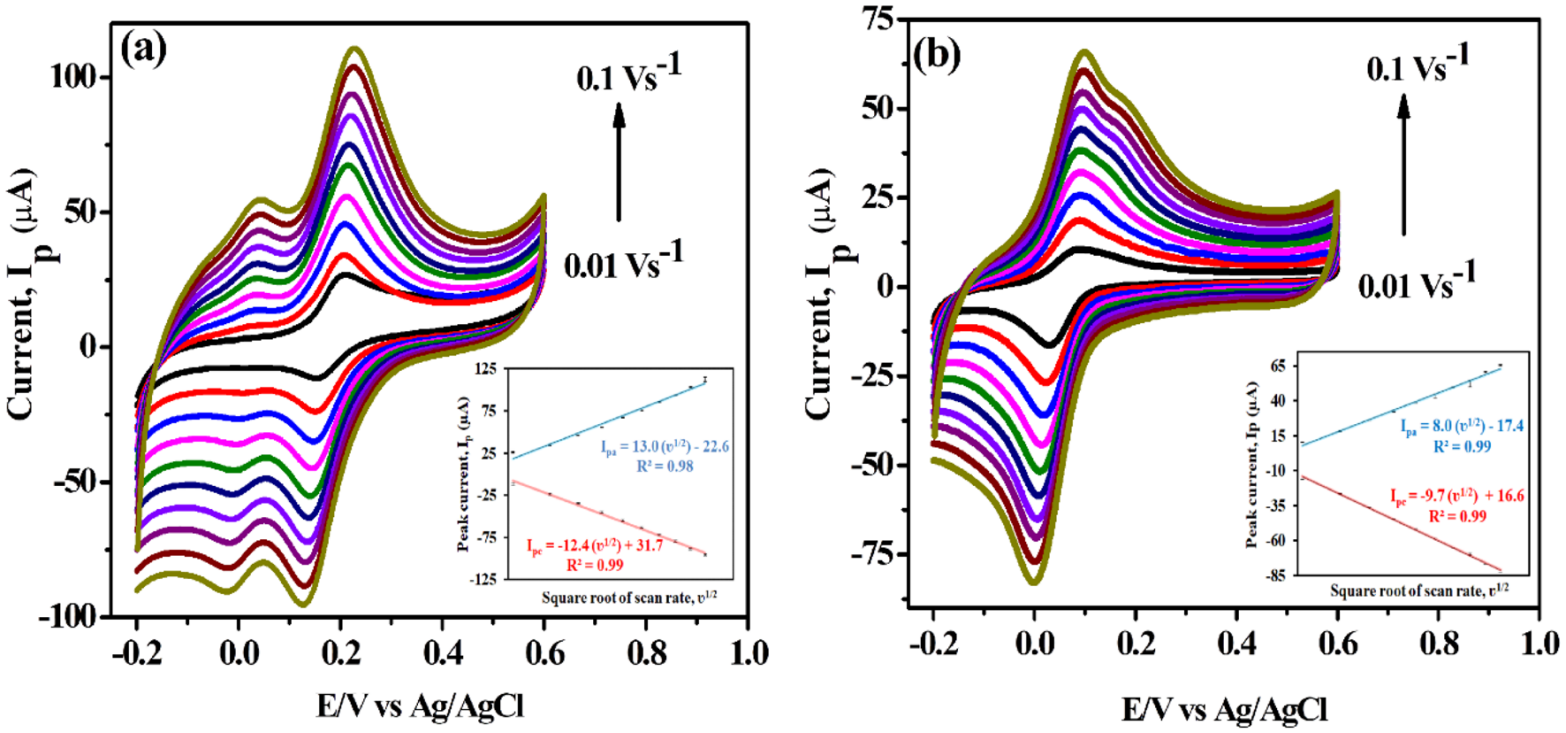
CV illustrating the effect of variable scan rates (0.01–0.1 Vs^−1^) on (**a**) 500 µM catechol and (**b**) 500 µM hydroquinone in 0.1 M PBS at CNCs/Zn-TPP/GCE electrode; Insets: Calibration plot of I_p(a,c)_ vs. square root of scan rate (ʋ^1/2^) for CC and HQ.

The electrochemical behavior of the 500 µM catechol and 500 µM hydroquinone (0.1 M PBS pH = 7.0) at CNCs/Zn-TPP/GCE was further investigated by changing the scan rate in the range of 0.01–0.1 Vs^−1^ as depicted in Fig. 8a,b. As the scan rate is increased a subtle increase in the redox peak currents was obtained. Similarly, the linear trend in the increase of redox peak current was observed when plotted against the square root of the scan rate and it is evident that the electro-redox process of both species at modified electrode is a diffusion-controlled mode. Herein the reason for the diffusion controlled mechanism is the large surface area of the composite material that accelerates the shuttling of charges between the analyte and the surface of the electrode and eventually high peak current occurs^59^. The plot for the regression equation in inset of Fig. 8a for both I_p(a,c)_ vs ʋ^1/2^ for catechol tells about the slope obtained and the regression equation is given as:

$$\begin{aligned} {\text{I}}_{{{\text{pa}}}} & = 13.01 \,\upupsilon ^{1/2} - \, 22.62 \left( {{\text{R}}^{2} = \, 0.99} \right) \\ {\text{I}}_{{{\text{pc}}}} & = \, - 12.46 \,\upupsilon ^{1/2} + \, 31.70 \, \left( {{\text{R}}^{2} = \, 0.99} \right) \\ \end{aligned}$$

Similarly, the regression equation obtained for hydroquinone (shown in Fig. 8b inset) is as follow:

$$\begin{aligned} {\text{I}}_{{{\text{pa}}}} & = \, 8.03 \,\upupsilon ^{1/2} {-} \, 17.49 \, \left( {{\text{R}}^{2} = \, 0.99} \right) \\ {\text{I}}_{{{\text{pc}}}} & = \, - 9.79 \,\upupsilon ^{1/2} + \, 16.61 \, \left( {{\text{R}}^{2} = \, 0.99} \right) \\ \end{aligned}$$

According to the Randles–Sevcik equation, it is concluded that if the slope of the plot of I_p_ vs log of scan rate is close to 0.5 or less than 1 then the electro-redox is a diffusion-controlled process. Here again we can see from the calibration plot with a linear regression equation for CC and HQ as:

$$\begin{aligned} \log \, \,{\text{I}}_{{{\text{pa}}}} \left( {CC} \right) \, & = \, 0.6 \, \log \, \,\upupsilon \, + \, 0.7 \, \left( {{\text{R}}^{2} = \, 0.98} \right) \\ \log \,{\text{ I}}_{{{\text{pa}}}} \left( {HQ} \right) \, & = \, 0.7 \, \log \, \,\upupsilon \, + \, 0.2 \, \left( {{\text{R}}^{2} = \, 0.99} \right) \\ \end{aligned}$$

the value of slope i.e., 0.6 and 0.7 (Fig. S5a,b) is less than 1^60^, therefore it is suggested that detection of catechol and hydroquinone is diffusion controlled process.

The electrochemical determination of catechol and hydroquinone is a reversible two-electron process which can be further justified by the following expression:

$$\Delta {\text{Ep}} {\text{E}}_{{{\text{pa}}}} {-}{\text{E}}_{{{\text{pc}}}} = \, \left( {59/{\text{n}}} \right){\text{ mV}}\,{\text{ at }}\,25\,^{ \circ } {\text{C }}$$

where E_pa_, E_pc_ and n are the anodic potential, cathodic peak potential and number of electrons involved in the system, respectively. Hence the electron count (n) obtained for CC is 1.79 and for HQ it is 2.3 that approximates to 2. The two electron redox mechanisms of these two analytes is supported from literature as well^61,62^.

### Concentration studies of catechol and hydroquinone using cyclic voltammetry (CV) and differential pulse voltammetry (DPV)

**Figure 9:**
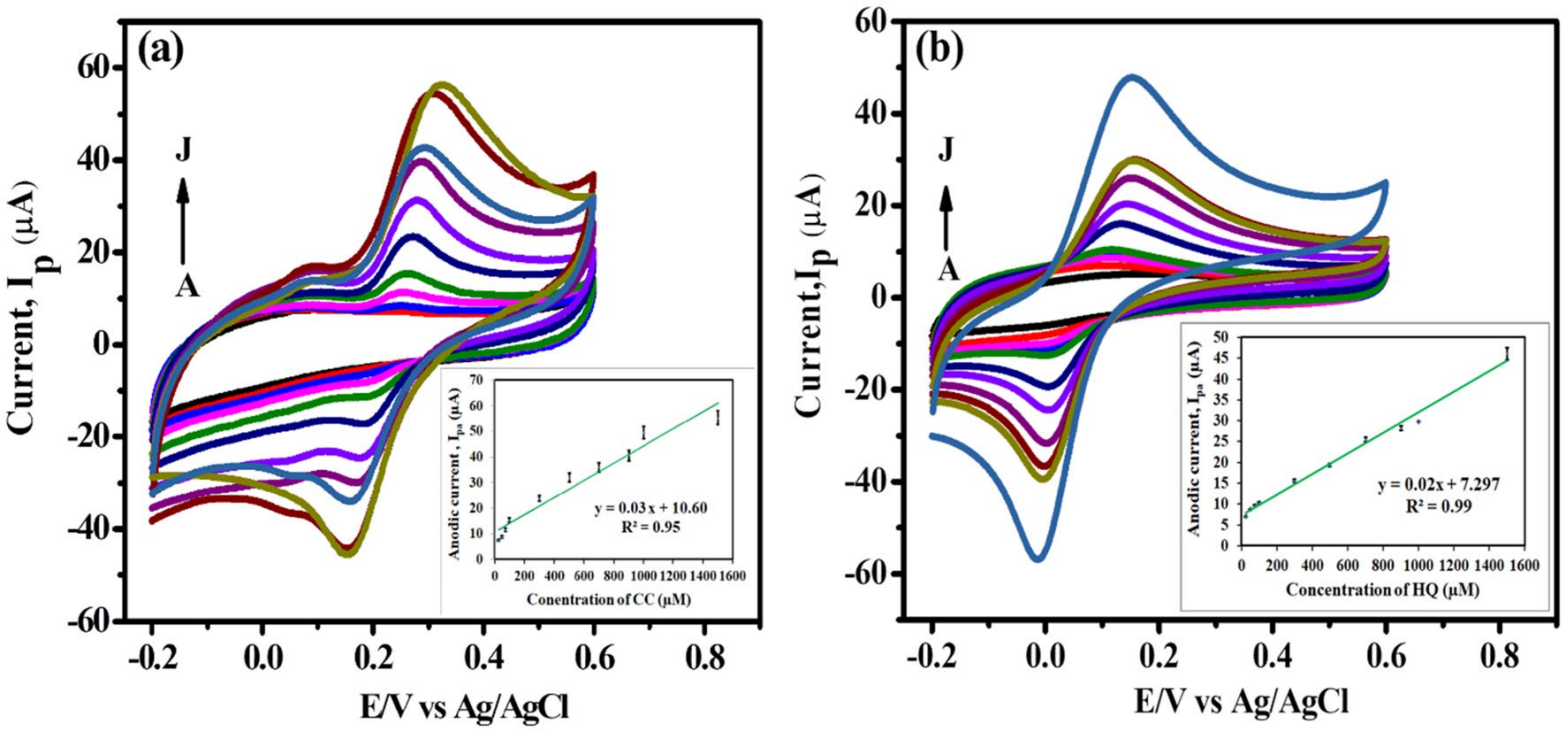
CV illustrating the effect of variable concentrations of (**a**) CC (A to J: 25, 50, 75, 100, 300, 500, 700, 900, 1000 and 1500 µM) and (**b**) HQ (A to J: 25, 50, 75, 100, 300, 500, 700, 900, 1000 and 1500 µM) in pH 7 (0.1 M) PBS solutions at CNCs/Zn-TPP/GCE nanosensor. (Insets: Calibration curve between different concentrations of catechol and HQ vs. anodic peak current).

Cyclic voltammetry was also performed to evaluate the individual and combined effects of different concentrations of CC and HQ on the peak current density using modified electrode, shown in Fig. 9a,b. From the cyclic voltammograms it can be seen as the concentration of catechol (Fig. 9a) and hydroquinone (Fig. 9b) is increased from 25 to 1500 µM, a gradual enhancement in the current is observed that exhibits the diffusion of electroactive species increases at the electrode surface and more current is detected^63^. It can also be seen that as the diffusion layer increases at higher concentration the broadening of peak takes place. Cyclic voltammograms demonstrate that the linear range, limit of detection, limit of quantification and sensitivity for catechol is between 25 and 1500 µM (R^2^ = 0.95), 0.9 µM, 3.1 µM and 0.48 µA µM^−1^ cm^−2^, respectively (Fig. 9a).

The linear range, limit of detection, limit of quantification and sensitivity for hydroquinone are 25–1500 µM (R^2^ = 0.99), 1.5 µM, 5.1 µM and 0.35 µA µM^−1^ cm^−2^, respectively, refer to Fig. 9b.

$$\begin{aligned} & {\text{CC: I}}_{{{\text{pa}}}} \left( {\upmu {\text{A}}} \right)_{{}} = \, 0.03 \, \,{\text{C }}\left( {\upmu {\text{M}}} \right) \, + \, 10.60 \, \left( {{\text{R}}^{2} = 0.95} \right) \\ & {\text{HQ: I}}_{{{\text{pa}}}} \left( {\upmu {\text{A}}} \right)_{{}} = \, 0.02 \, \,{\text{C }}\left( {\upmu {\text{M}}} \right) \, + \, 7.29 \, \left( {{\text{R}}^{2} = 0.99} \right) \\ \end{aligned}$$

**Figure 10:**
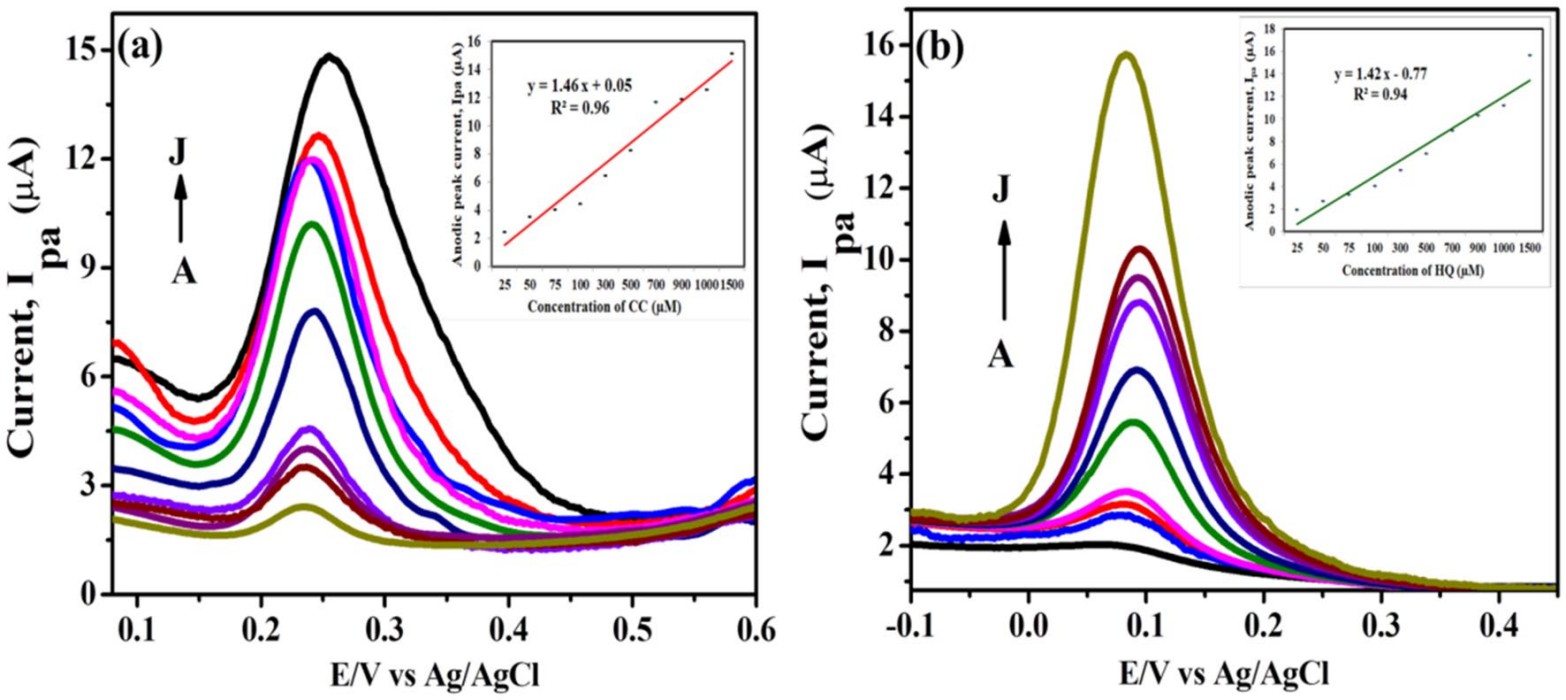
(**a**) DPV illustrating the effect of individual concentrations of (**a**) CC (A to J: 25, 50, 75, 100, 300, 500, 700, 900, 1000 and 1500 µM) in 0.1 M PBS at CNCs/Zn-TPP/GCE electrode and (**b**) HQ (A to J: 25, 50, 75, 100, 300, 500, 700, 900, 1000 and 1500 µM). Insets: Calibration curve between different concentrations of analytes vs. anodic peak current.

Further DPV for individual detection of the compounds was performed (shown in Fig. 10a,b) to analyze the sensitivity of the electrode system towards both the analytes and the voltammograms support the results of CV in aspect that the material sensed CC and HQ at separate well-defined potentials and as the concentration of respective analytes is increased, the gradual increase in the current intensity takes place which reveals the efficient and fast diffusion of electroactive species at the interface.

The regression equations for DPV studies of CC and HQ are given below:

$$\begin{aligned} & {\text{CC: I}}_{{{\text{pa}}}} \left( {\upmu {\text{A}}} \right) = \, 1.46 \, \,{\text{C }}\left( {\upmu {\text{M}}} \right) \, + \, 0.05 \, \left( {{\text{R}}^{2} = 0.96} \right) \\ & {\text{HQ: I}}_{{{\text{pa}}}} \left( {\upmu {\text{A}}} \right) = \, 1.42 \, \,{\text{C }}\left( {\upmu {\text{M}}} \right) \, + \, 0.77 \, \left( {{\text{R}}^{2} = 0.94} \right) \\ \end{aligned}$$

### Simultaneous detection in binary mixtures of catechol and hydroquinone

The simultaneous detection studies were done using binary mixture containing CC (50, 75, 100, 300, 500, 700 and 900 µM) in the presence of 500 µM HQ and HQ (50, 75, 100, 300, 500, 700 and 900 µM) in the presence of 500 µM CC as shown in Fig. S6a,b, respectively. From the above individual analysis of both the analytes it can be deduced that these isomeric species can also be conveniently detected in a binary mixture. Cyclic voltammograms and differential pulse voltammograms show the well separation of the peak potential of both the isomers with a ∆E_pa(HQ-CC)_ of 110 mV. It is concluded that the proposed novel nanosensor exhibits the best catalytic activity for the simultaneous estimation of the two isomers. CNCs/Zn-TPP/GCE efficiently and selectively detects the two positional isomers that usually co-exist in nature because the peaks are not overlapped^64^.

### Selectivity, repeatability and stability of CNCs/Zn-TPP/GCE modified electrode isomeric interference

The effect of resorcinol (1,3-dihydroxybenzene), as an interfering species was evaluated that lie closer to catechol and hydroquinone in the potential window where CC and HQ are detected. It is noteworthy that no significant change in the detection of CC and HQ was observed upon addition of interfering entity. Resorcinol (RS), the positional isomer to CC and HQ, exhibited no interference and detected at more positive oxidation potential approximately near to 0.6 V that articulates the less reactive nature of aromatic OH groups present at meta to each other in RS whereas catechol (ortho -OH groups) and hydroquinone (para -OH groups) are detected earlier at 0.2 V and 0.1 V, respectively. CC and HQ were shown to be more reactive due to the position of OH groups contributing towards more resonating structures and increased conjugated behavior of the aromatic structure thus enhancing the rate of release of protons in both the isomers. Therefore due to the active nature of CC and HQ these are diffused more conveniently to the surface of CNCs/Zn-TPP/GCE modified electrode and undergo redox reaction as compared to RS that is less activated and only oxidation peak is observed for it^65^. Figure S7 represents the results of DPV of ternary mixture containing all the three isomers i.e., 500 µM each of CC, HQ and RS which clearly indicates that these isomers appear at different potentials with well resolved peaks and high selectivity of fabricated electrode is observed.

#### Effect of other interferring species

DPV was performed to further analyze the interfering effect of different organic molecules and ions present in the solution we used threefold more quantities of these interfering species as compared to CC and HQ. The bar graph depicts that a minor change in current was observed when the interfering species were added to the solutions containing CC and the HQ (Fig. S8, Supplementary Information). The interfering species used for the analysis were ascorbic acid (AA), uric acid (UA), para-nitrophenol, resorcinol (RS), Cl^−1^, SO_4_^–2^, Mg^+2^, Co^+2^, Ca^+2^, K^+1^, and Na^+1^. This shows that the developed sensor can be applied for the practical application to detect the presence of environmental pollutants.

The electrode was subjected to eight repetitive measurements in 500 µM CC and 500 µM HQ separately (Fig. S9, Supplementary Information) and the relative standard deviation values found as 4.50% (CC) and 5.43% (HQ) as shown in Table S2 (Supplementary Information).

Similarly, the stability of the sensor towards 500 µM CC and 500 µM HQ was studied after 30 days of storage. The percentage loss in the initial activity of the sensor was 6.74% (CC) and 13.66% (HQ), as shown in Table S3 (Supplementary Information). Hence it depicts the good stability of the electrode.

The performance of the prepared nanosensor was further compared to the other nanosensors reported in the literature and it represents that the present nanosensor exhibits good performance as shown in Table 1. The table shows different type of electrodes used for the detection of CC and HQ so far and it can be observed that the CNCs/Zn-TPP/GCE has comparable results in some cases it has better performance in the sense of LOD when compared to Sr. No. 2 that exhibits 4 µM LOD for CC and that of HQ it is 0.6 µM, considering the use of electrode in Sr. No. 3 so again CNCs/Zn-TPP/GCE exhibits better results in terms of concentration range for CC and also it is used for the detection of both dihydroxybenzene isomers whereas Sr. No. 3 is used for CC detection. Furthermore, it has better electrochemical activity as compared to electrodes in Sr. No. 4 and Sr. No. 5 in terms of LOD and linear concentration ranges. While some other reported electrodes in the Table 1 show better performance than CNCs/Zn-TPP/GCE like Sr. No. 8, Sr. No. 7 but in these cases precious metals and the sophisticated methods of preparation are used. However, CNCs/Zn-TPP nanocomposite does not require any sophisticated methodology, it is cost effective and stable at room temperature up to 30 days.

**Table 1:** Comparison of performance of CNCs/Zn-TPP/GCE for catechol (CC) and hydroquinone (HQ).

S. no	Electrode	Limit of detection (LOD)		Linear range		References
		CC	HQ	CC	HQ	
1	ECF-CPE^a^	0.2 µM	0.4 µM	1–200 µM	1–200 µM	^66^
2	MIL-101 (Cr)-rGO^b^	4 µM	0.66 µM	10–1400 µM	4–1000 µM	^67^
3	EGr-TPyP/GC^c^	3.3 × 10^–7^ M	–	10^–6^–10^–4^ M	–	^65^
4	CdTe QDs/GR^d^	18.28 µM	–	30–1000 μM	–	^68^
5	Co_3_O_4_/MWCNTs/GCE	8.5 μM	5.6 μM	10–700 μM	10–800 μM	^69^
6	MWCNTs/p-DAN/GCE^e^	1.0 × 10^−8^ M		2.0 × 10^−8^–1.3 × 10^−4^ M		^70^
7	Co3O4@carbon core/shell nanostructure	0.03 μM	0.03 μM	0.6–116.4 μM	0.8 – 127.1 μM	^71^
8	GO@PDA–AuNPs/GCE^f^	15 nM	–	0.3–67.55 μM	–	^72^
9	CNCs/Zn-TPP/GCE^g^	0.9 μM	1.5 μM	25–1500 µM	25–1500 µM	Present work

^a^ECF-CPE: Electrospun Carbon Nanofiber-Carbon paste electrode.

^b^MIL-101 (Cr)-rGO: Metal organic framework-reduced graphene oxide.

^c^EGr-TPyP/GC: Electrochemically exfoliated graphene-tetrapyridylporphyrin/Glassy carbon electrode.

^d^CdTe QDs/GR: Cadmium Telluride Quantum dots/Graphene.

^e^MWCNTs/p-DAN/GCE: Multi-walled carbon nanotubes/poly(1,5-diaminonaphthalene) composite film modified electrode.

^f^GO@PDA–AuNPs/GCE: Gold nanoparticles decorated graphene oxide@polydopamine composite.

^g^CNCs/Zn-TPP/GCE: Carbon Nanocoils/Zinc-tetraphenylporphyrin/Glassy carbon electrode.

### Real sample analysis

Real sample analysis was performed to analyze the practical application of the proposed sensing material and the standard addition protocol was employed. According to the protocol in two different flasks tap water samples were diluted with phosphate buffer (pH 7) then 300 µM CC was added in one flask and 300 µM HQ in the other flask. The resulted solutions were analyzed by CV. The recovery of CC and HQ was 123% and 90%, respectively (Table 2). The results show that the prepared nanosensor can be used for practical application to detect environmental pollutants present in industrial wastes etc.

**Table 2:** Detection of both CC and HQ in tap water.

Tap water sample	Added (µM)	Found (µM)	Recovery (%)
Catechol	300	370	123
Hydroquinone	300	271	90

## Conclusions

In this work, a novel nanosensor CNCs/Zn-TPP/GCE was formulated to electrochemically sense catechol and hydroquinone individually and simultaneously in binary mixtures. CNCs/Zn-TPP/GCE showed good sensing ability as well as well resolved anodic peaks of CC and HQ with a peak potential separation of both isomers as ∆E_pa (CC-HQ)_ of 110 mV. A wide linear range obtained for catechol was 25–1500 µM with LOD of 0.9 µM, LOQ as 3.1 µM and sensitivity as 0.48 µA µM^−1^ cm^−2^, respectively. Similarly, the modified electrode showed linear response range of 25–1500 µM for hydroquinone with LOD, LOQ and sensitivity of 1.5 µM, 5.1 µM and 0.35 µA µM^−1^ cm^−2^, respectively. Further the interference studies were performed at CNCs/Zn-TPP/GCE electrode with resorcinol and no interference was observed in the detection of CC and HQ. It is suggested that this novel nanosensor can be further used for the detection of catechol and hydroquinone in real samples.

## Methodology

### Reagents and instrumentation

The chemicals used in the present study i.e., zinc acetate, silica gel (for chromatography), hydroquinone (99.5%) and n-hexane were procured from Sigma-Aldrich and benzaldehyde (≥ 99%), catechol (99%), resorcinol (99%), chloroform (for analysis), n-hexane, dimethyl formamide (DMF) (99.5%), hydrochloric acid, sodium hydroxide, potassium chloride (≥ 99%), potassium ferricyanide (99%), potassium dihydrogen phosphate (≥ 99%) and dipotassium hydrogen phosphate (≥ 99%) were purchase from Merck. Pyrrole (96%) was purchased from Fluka, ethanol (95–97%) was of Pak Made and propionic acid (99%) was of Acros Organics. All these chemicals were used as such and of analytical grade, only pyrrole was distilled before use. Carbon nanocoils were obtained from Dalian University of Technology, China. For the polishing of glassy carbon electrode two different polishing suspensions i.e., diamond polish (1 µm) and alumina polish (0.05 µm) were obtained from Gamry Instruments.

Electrochemical measurements were recorded with the high performance potentiostat used was Gamry Instrument Reference 3000/3000 AE/Potentiostat/Galvanostat/ZRA (USA) using a three electrode-cell system. The reference electrode, counter electrode and working electrodes used were of Ag/AgCl, platinum wire and nanocomposite deposited on glassy carbon electrode, respectively. 3 M KCl (saturated) solution in deionized water was used as a filling solution for the Ag/AgCl reference electrode. The surface area of the working electrode (WE) was 0.07 cm^2^. The measurements were carried out in a potential window of – 0.2 and + 0.6 V (vs. Ag/AgCl) with a sweep rate of 0.05 Vs^−1^. Moreover, morphology of the prepared composite was determined using Mira3 Tescan scanning electron microscope (SEM) at a working voltage of 20 kV and energy dispersive X-ray spectroscope (EDS) was used for analysis of elements present in the nanocomposite. Bruker Alpha platinum-ATR was employed to perform Fourier transform infrared spectroscopy (ATR-FTIR). For UV–visible analysis the spectra were recorded with Perkin Elmer Lambda 365 inthe wavelength range of 200–800 nm.

### Synthesis of 5,10,15,20-tetraphenylporphyrin (TPP)

The Adler-Longo protocol was followed for the synthesis of TPP^73–75^. For the synthesis of TPP, propionic acid (273 mL) was refluxed with the addition of freshly distilled pyrrole (72.4 mmol; 5 mL) and benzaldehyde (68.60 mmol; 7 mL). After the addition of the reactants the reaction mixture was further refluxed for 30 min. Then the mixture was cooled to room temperature, filtered, washed with ethanol and dried in vacuum oven at 50 °C. The impurities were removed by column chromatography to obtain pure purple crystals of TTP (yield: 21.8%).

### Synthesis of zinc-5,10,15,20-tetraphenylporphyrin (Zn-TPP)

The above synthesized tetraphenylporphyrin (110 mg) was dissolved in chloroform (solution A) and zinc acetate tetrahydrate (330 mg) was dissolved in methanol (solution B). Both the solutions A and B were mixed in a round bottom flask and refluxed for 2 h. Then the reaction mixture was cooled to room temperature, and the solvent was evaporated by rotary evaporator. The product (Zn-TPP) was further purified by column chromatography (yield 87%)^76^.

### Preparation of CNCs/Zn-TPP nanocomposite

The nanocomposite was prepared using sonochemical method with certain modifications^77^. For the preparation of CNCs/Zn-TPP, the solution of Zn-TPP (10 mg/10 mL DMF) was added to the CNCs suspension (5 mg/5 mL DMF), the suspended mixture was then stirred (12 h) and ultrasonicated for 3 h at room temperature (RT), centrifuged (6000 rpm; 20 min), washed with ethanol and dried under vacuum at 60 °C for 24 h. CNCs/TPP nanocomposite was also prepared through sonochemical method for comparative analysis of metallated and non-metallated TPP.

### Protocol for electrode preparation

The nanosensor electrode was prepared by simple drop casting the nanocomposite material onto the surface of pre-polished glassy carbon electrode^78,79^. Two types of polishing suspensions have been used for the GCE i.e. diamond polish and alumina polish. Diamond polishing was carried out by placing the diamond pad on the holding stand. 2 drops of diamond polish were spread over the diamond pad, GCE was held vertically and moved continuously over the pad in figure-eight motion and then rotated at 90° angle after every figure-eight cycle for even polishing of electrode. GCE was then rinsed with de-ionized water followed by ultrasonication in ethanol, acetone and de-ionized H_2_O sequentially for 5 min each and air dried. The working electrode was then polished using 0.05 μm ɤ-alumina slurry using the same method.

Afterwards GCE having 3 mm diameter was modified with composites for electrochemical sensing of CC and HQ. About 2 mg of CNCs/Zn-TPP nanocomposite was dispersed in 1 ml of DMF by applying bath sonication unless no agglomerates were observed and a homogeneous suspension (ink) result. 5 µl of the nanocomposite suspension was pipetted out and drop casted onto the mirror like surface of glassy carbon electrode. Then the electrode was dried in air at ambient temperature for electrochemical sensing of CC and HQ. The CNCs, TPP, Zn-TPP and CNCs/TPP inks were also prepared and coated on GCE in the same manner.

## Supplementary Information

Supplementary information.
