# Supplementary material for: Supramolecular assemblies of carbon nanocoils and tetraphenylporphyrin derivatives for sensing of catechol and hydroquinone in aqueous solution
Source: Sci Rep. 2021 Mar 3;11:5044. doi: 10.1038/s41598-021-84294-7 (PMC7930085; doi:10.1038/s41598-021-84294-7)
Supplement: Supplementary file 1 — Supplementary information. [file 41598_2021_84294_MOESM1_ESM.docx]

**Supplementary Data for**

# Supramolecular Assemblies of Carbon Nanocoils and Tetraphenylporphyrin Derivatives for Sensing of Catechol and Hydroquinone in Aqueous Solution

Syeda Aqsa Batool Bukhari^1^, Habib Nasir^1*^, Lujun Pan^2^, Mehroz Tasawar^1^, Manzar Sohail^1^, Muhammad Shahbaz^1^, Fareha Gul^1^ and Effat Sitara^1^

^1^ Department of Chemistry, School of Natural Sciences (SNS), National University of Sciences and Technology (NUST), H-12, Islamabad, Pakistan

^2^ School of Physics, Dalian University of Technology, Dalian, China

*EDS Analysis*


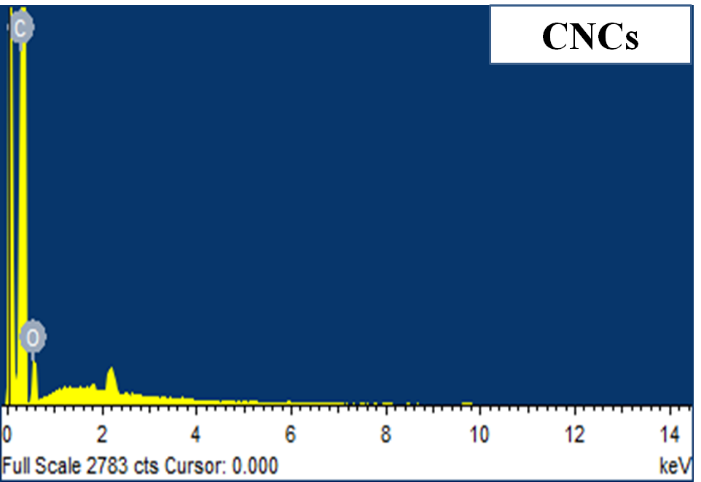


**Figure S1.** EDS of pure CNCs.


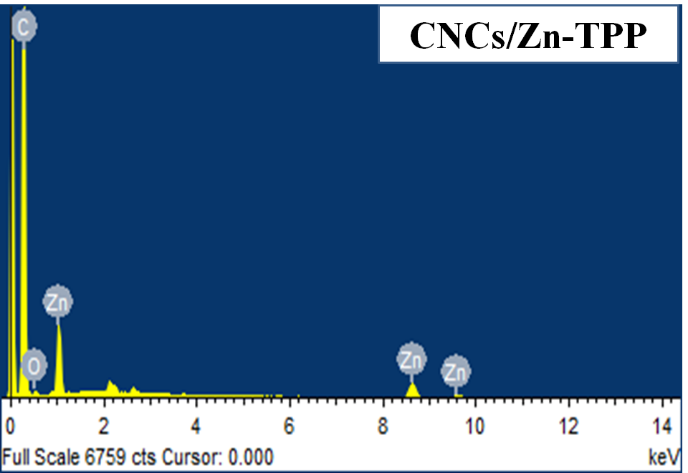


**Figure S2**. EDS of CNCs/Zn-TPP nanocomposite.

***EIS Studies***


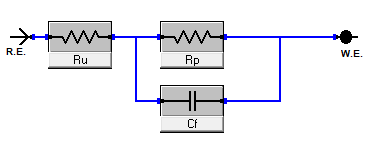


Where, R.E. =Reference electrode, R_u_ = Resistance of the solution, R_p_ = R_ct_ = Charge transfer resistance, C_f_ = Double layer capacitance and W.E. = Working electrode.

**Figure S3.** Randles Model as an equivalent circuit for EIS fitting.

***Mechanism for Catechol and Hydroquinone Sensing***


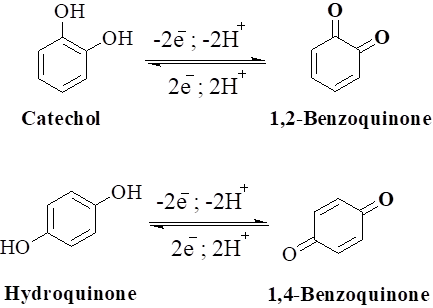


**Figure S4.** Probable mechanism for the electrochemical detection of CC and HQ

***Effect of Scan Rate***


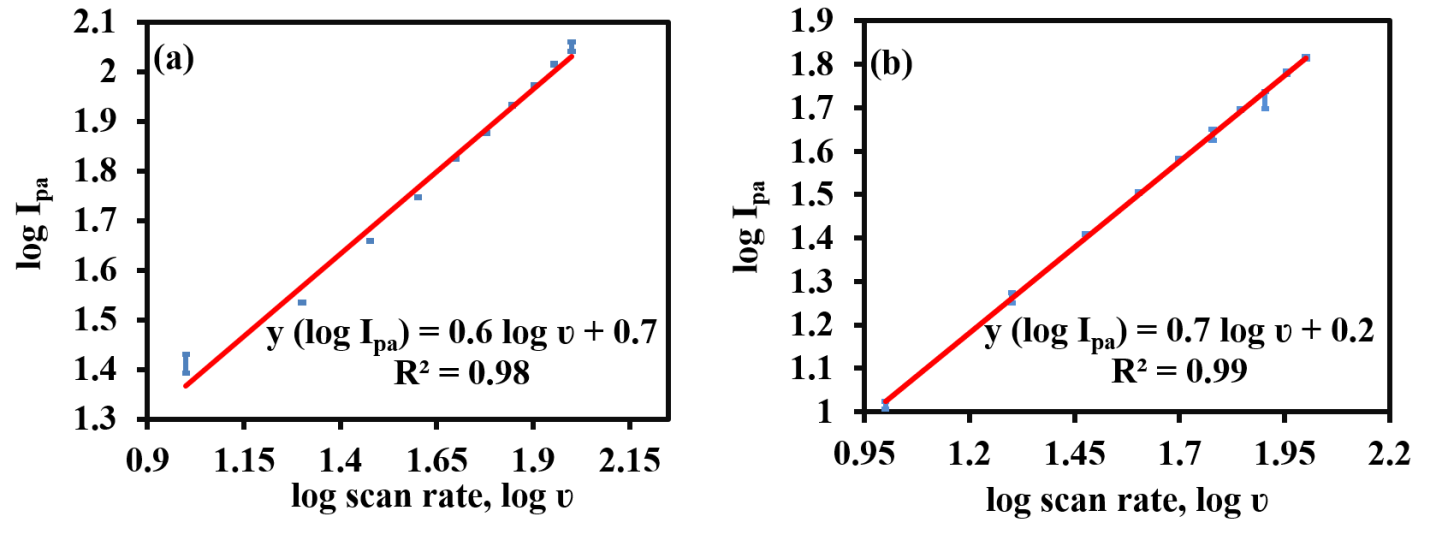
**Figure S5.** Calibration plot of log I_pa_ vs. log scan rate, log ʋ for (a) 500 µM catechol and (b) 500 µM hydroquinone.

***Simultaneous Detection in Binary Mixtures of Catechol and Hydroquinone***


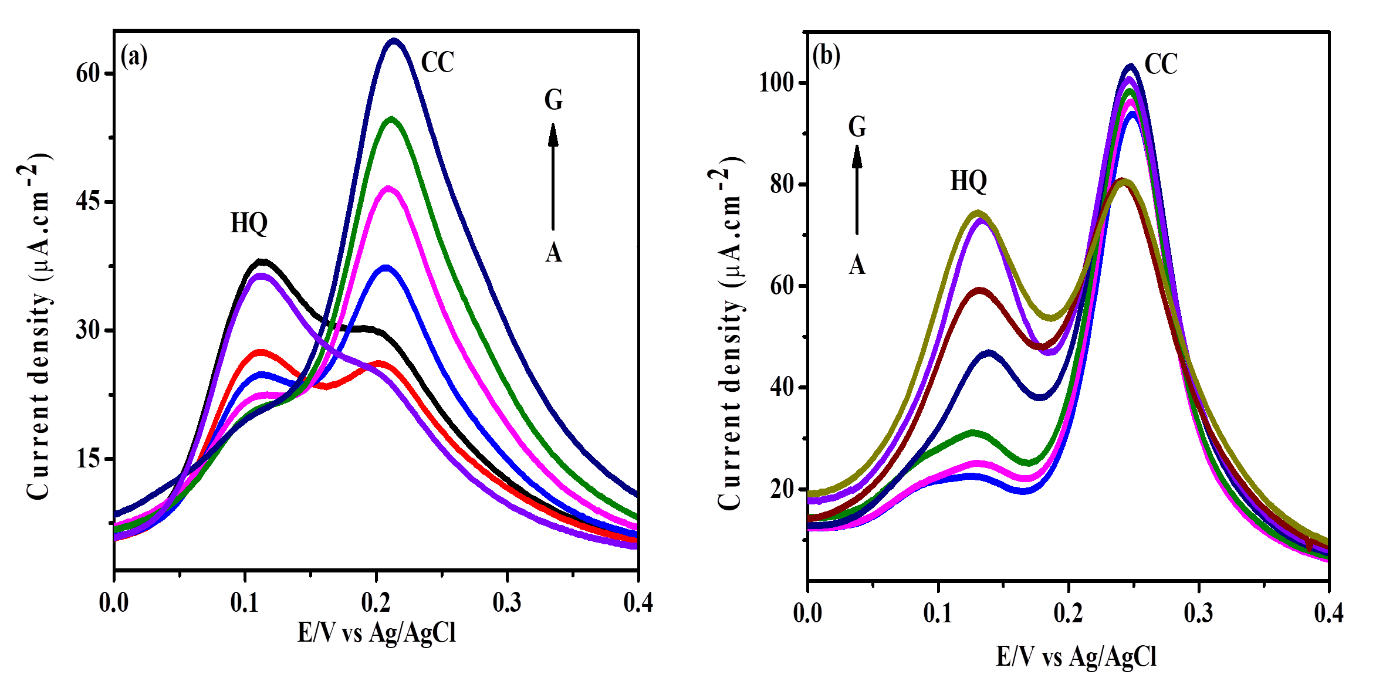


**Figure S6.** DPV illustrating the detection of (a) CC (A to G: 50, 75, 100, 300, 500, 700 and 900 µM) in the presence of 500 µM HQ; (b) HQ (A to G: 50, 75, 100, 300, 500, 700 and 900 µM) in the presence of 500 µM CC at CNCs/Zn-TPP/GCE fabricated nanosensor.

***Selectivity Studies***


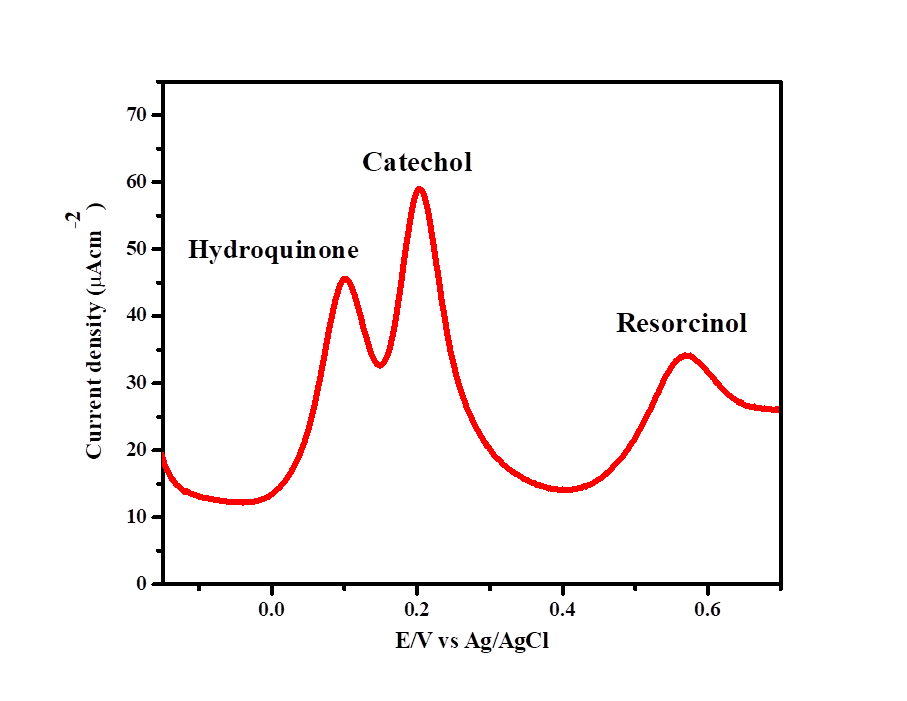


**Figure S7**. DPV illustrating the interference study of CC, HQ and RS at CNCs/Zn-TPP/ GCE electrode.


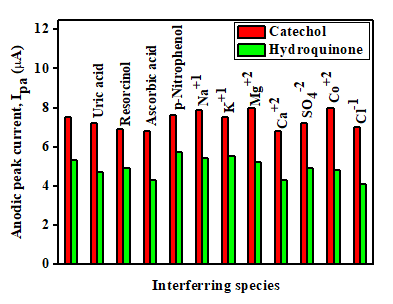


**Figure S8**. Bar graph illustrating the effect of organic and inorganic interfering species on the detection current of CC and HQ at CNCs/Zn-TPP/GCE nanosensor in 0.1 M PBS (pH=7; Scan rate= 0.025 Vs^-1^) using DPV.

*Repeatability Studies*


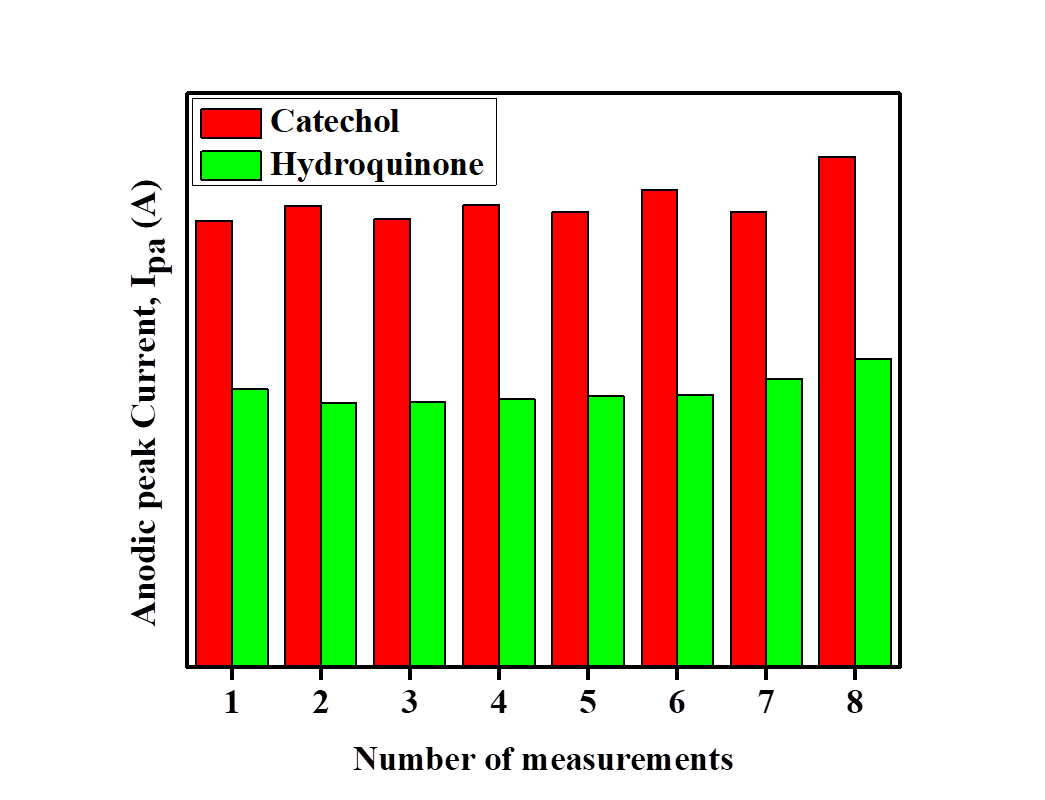


**Figure S9.** CV illustrating the repeatability studies of CNCs/Zn-TPP/GCE nanosensor in 500 µM catechol and 500 µM hydroquinone.

***Tables***

**Table S1**. Electrochemical surface area (ESA) of electrodes

| Electrodes | ESA (cm^2^) |
| --- | --- |
| GCE | 0.19 |
| Zn-TPP | 0.07 |
| CNCs | 0.26 |
| CNCs/Zn-TPP/GCE | 0.23 |

**Table S2.** Data for relative standard deviation (RSD%) obtained for eight repetitive experiments done for CC and HQ at CNCs/Zn-TPP/GCE electrode.

| Analyte | Concentration | Anodic Peak Current, I_pa_ (µA) | Average Current | Standard Deviation | RSD % |
| --- | --- | --- | --- | --- | --- |
|  |  | **(Repetitions)** |  |  |  |
| Catechol | 500 µM | 32.67 | 34.02 | 1.53 | 4.50% |
|  |  | 32.85 |  |  |  |
|  |  | 33.78 |  |  |  |
|  |  | 33.8 |  |  |  |
|  |  | 33.34 |  |  |  |
|  |  | 33.34 |  |  |  |
|  |  | 34.96 |  |  |  |
|  |  | 37.38 |  |  |  |
| Hydroquinone | 500 µM | 20.36 | 20.28 | 1.10 | 5.43% |
|  |  | 19.33 |  |  |  |
|  |  | 19.4 |  |  |  |
|  |  | 19.61 |  |  |  |
|  |  | 19.87 |  |  |  |
|  |  | 19.94 |  |  |  |
|  |  | 21.11 |  |  |  |
|  |  | 22.6 |  |  |  |

*Stability Studies*

Table S3. Data for % activity loss of CNCs/Zn-TPP/GCE electrode towards CC and HQ detection after storage for 30 days.

| **Analyte** | **Concentration**  **(µM)** | **Anodic Peak Current, I_pa_ (µA)** | | **% Loss of Electrode Activity** |  |
| --- | --- | --- | --- | --- | --- |
|  |  | **Initial Activity** | **After 30 days**  **of storage** |  | |
| Catechol | 500 µM | 34.53 | 32.20 | 6.74% | |
| Hydroquinone | 500 µM | 18.16 | 15.68 | 13.66% | |
